# Music as an Intervention to Improve the Hemodynamic Response of Ketamine in Depression: A Randomized Clinical Trial

**DOI:** 10.1001/jamanetworkopen.2023.54719

**Published:** 2024-02-05

**Authors:** Kyle T. Greenway, Nicolas Garel, Lê-Anh L. Dinh-Williams, Serge Beaulieu, Gustavo Turecki, Soham Rej, Stephane Richard-Devantoy

**Affiliations:** 1 Department of Psychiatry, Faculty of Medicine, McGill University, Montréal, Quebec, Canada; 2 Lady Davis Institute, Jewish General Hospital, Montréal, Quebec, Canada; 3 Douglas Mental Health University Institute, Montréal, Québec, Canada; 4 Douglas Mental Health Research Institute, McGill Group for Suicide Studies, Montréal, Québec, Canada; 5 McGill Meditation and Mind-Body Medicine Research Clinic and Geri-PARTy Research Group, Lady Davis Research Institute and Jewish General Hospital, Montreal, Quebec, Canada

## Abstract

This randomized clinical trial explores whether music improves the hemodynamic response of ketamine among patients with treatment-resistant depression in Canada.

## Introduction

Subanesthetic ketamine is emerging as 1 of the most effective interventions for treatment-resistant depression (TRD).^1^ Ketamine’s stimulatory hemodynamic effects are among its most important adverse effects—up to 20% of patients receiving intravenous (IV) ketamine for TRD require pharmacological management of treatment-induced hypertension.^1^ Music has previously been found to improve the hemodynamic response of other medical interventions but has not been explored for ketamine in TRD.^2^

## Methods

The Music for Subanesthetic Infusions of Ketamine (MUSIK) randomized clinical trial was conducted between January 2021 and August 2022 in Montreal, Canada, and approved by the ethics committees of the Douglas Mental Health University Institute and the Jewish General Hospital (NCT04701866). Participants had a current unipolar or bipolar depressive episode refractory to at least 2 adequate medication trials, were accepted for treatment by IV ketamine, and provided written informed consent. Race was self-reported by study participants as a standard part of their clinical psychiatric assessments. This randomized clinical trial followed the CONSORT reporting guideline.

The trial protocol, including full inclusion and exclusion criteria, can be found in Supplement 1. Patients were randomly assigned in a 1:1 ratio to receive curated music throughout their 40-minute ketamine infusions (intervention), or usual care (no music; control), during a course of 6 subanesthetic doses (0.5 mg/kg) over 4 weeks.

The primary outcome was the change in systolic blood pressure (BP) between intervention and control groups from baseline (immediately before infusion commencement) to its termination. The change in systolic BP was operationalized as the difference between mean triplicate measurements at 0 and 40 minutes of each infusion. Forty minutes was prospectively selected as the key time point as it corresponds to peak plasma values.

Generalized estimating equations were used for mean population effect estimates of treatment conditions (music vs no music) on changes in systolic and diastolic BP across all 6 ketamine infusion sessions, accounting for clustering of the longitudinal and repeated measures.^3^ Analyses used an intention-to-treat approach with inverse-probability weighting for missing data, and 2-tailed *P* < .05 was considered statistically significant.^4^ The study protocol (Supplement 1) and supplemental methods (eMethods, eTable 1, and eTable 2 in Supplement 2) provide further analysis details, including the sample size calculation, which estimated 30 patients to be sufficient. Analyses were conducted from September 2022 to July 2023 using R version 4.02 (R Project for Statistical Computing).

## Results

**Figure 1.**
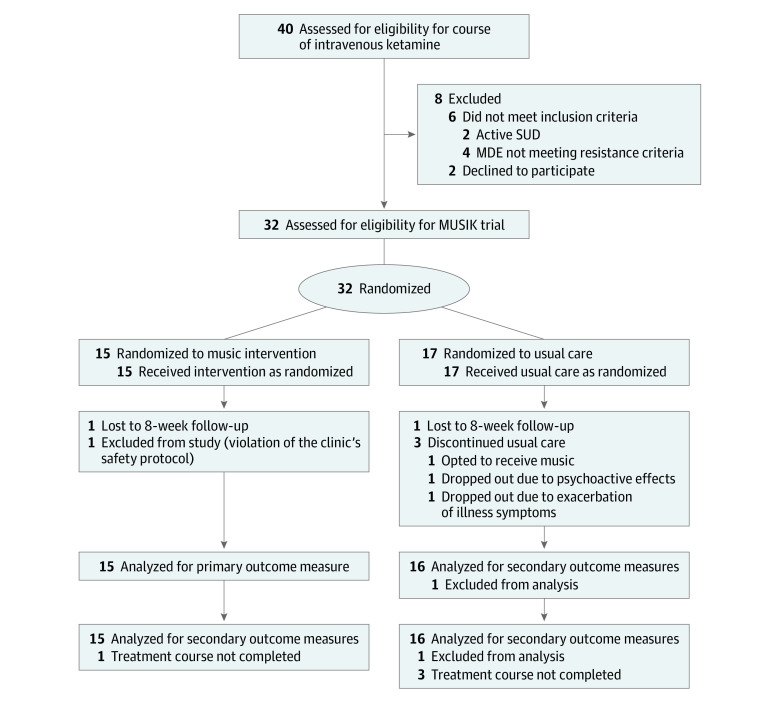
Study Flow Diagram MDE indicates major depressive episode; MUSIK, Music for Subanesthetic Infusions of Ketamine randomized clinical trial; SUD, substance use disorder.

Thirty-two patients were enrolled; 15 in the music group and 17 in the nonmusic group (Figure 1). Of these, 23 (72%) were women, and the mean (SD) age was 45.9 (14.2) years; 1 (3%) was Asian; 1 (3%) was Black, 2 (6%) were Hispanic, 1 (3%) was Middle Eastern, and 27 (84%) were White. No serious adverse events were recorded, and 28 patients completed the trial per protocol (Figure 1).

**Figure 2.**
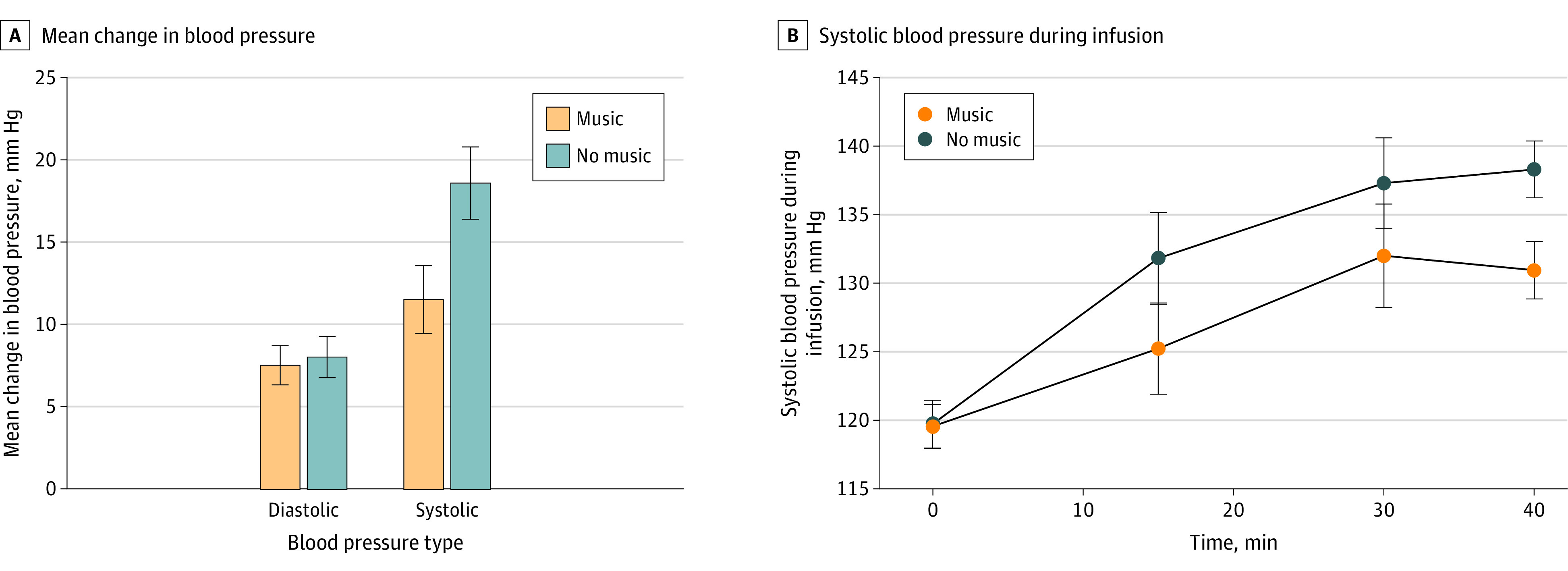
Aggregate Group Changes in Blood Pressure Figure shows aggregate group changes in blood pressure across 181 ketamine infusions: differences from baseline to 40 minutes for systolic and diastolic blood pressure calculated using generalized estimating equations (A), and the raw mean systolic blood pressure values over the course of the infusion (B). Error bars indicate 95% CIs.

A total of 181 ketamine infusions were administered (87 music; 94 nonmusic). Mean population effect estimates of conditions found that music significantly reduced increases in systolic BP (β = −7.4 mm Hg [95% CI, −12.0 to −2.9 mm Hg]; *P* = .008), but not diastolic BP (β = −0.8 mm Hg [95% CI, −1.59 to 3.18 mm Hg]; *P* = .58), compared with the control (Figure 2).

## Discussion

The MUSIK trial found that music significantly decreased ketamine-induced systolic BP changes. Music may thus represent a feasible, benign, nonpharmacological approach to improving the hemodynamic response of subanesthetic ketamine infusions for TRD. Secondary trial outcomes will be published elsewhere.

Beyond psychiatric contexts, the stimulatory hemodynamic effects of ketamine have received extensive study, both as desirable (eg, in surgical contexts) and as potentially harmful (eg, in procedural sedation),^5^ but never in relation to music, to our knowledge. The magnitude of the hemodynamic effects observed in this study suggests that ketamine research should consider potential influences of contextual factors, including auditory stimuli. Ketamine’s unique psychoactive effects may amplify the effects of music and other environmental influences, as has been long observed in the anesthesiology literature.^6^

Although the MUSIK trial administered 181 ketamine infusions, its modest sample size is a limitation. Further investigation is needed in larger samples and additional clinical contexts.

## Notes

Supplement 1.
Trial Protocol and Statistical Analysis Plan

Supplement 2.
eMethods
**eTable 1.** Baseline Demographics and Clinical Characteristics **eTable 2.** Musical Playlists Employed in the Study
eReferences

Supplement 3.
Data Sharing Statement
