# Supplementary material for: Music as an Intervention to Improve the Hemodynamic Response of Ketamine in Depression: A Randomized Clinical Trial
Source: JAMA Netw Open. 2024 Feb 5;7(2):e2354719. doi: 10.1001/jamanetworkopen.2023.54719 (PMC10845001; doi:10.1001/jamanetworkopen.2023.54719)
Supplement: Supplement 1. — Trial Protocol and Statistical Analysis Plan [file jamanetwopen-e2354719-s001.pdf]

This supplement contains the following items:

1. Original protocol, final protocol, summary of changes.

2. Original statistical analysis plan, final statistical analysis plan, summary of changes.

PROTOCOL

Version: 5.0

Version Date: March 2nd, 2022

Original Protocol Date: 20 November 2020

Study code: DOUGKMT01

Music as an Intervention to Improve the Hemodynamic Tolerability of Ketamine in Depression: a pilot randomized control trial.

Table of contents

Version history and summary of changes 5

Protocol summary ………………………………………………………………………..........8

1. Background and rationale 11

2. Study objectives..13

2.1 Primary objective...13

2.2 Secondary objectives 13

2.3 Exploratory objective..13

3. Study design (study amendment version 5.0)...14

4. Study population..16

4.1 Inclusion criteria ……………………………….. 16

4.2 Exclusion criteria..17

4.3 Antihypertensive medication..19

4.4 Randomization procedures...19

4.5 Participant withdrawal or discontinuation from study....................................19

5. Study endpoints..20

5.1 Primary outcome measure...20

5.2 Secondary outcome measures (study amendment version 2.0, 3.0 and 4.0) 20

6. Study intervention and control groups..21

6.1 Music during ketamine treatment…………………………………………………21

6.2 Control condition: treatment as usual……………………………………….…23

7. Study procedures and guidelines 23

7.1 Clinical assessments...23

7.1.1 Demographics, medical history, and medication 23

7.1.2 Blood pressure and hemodynamic function 24
7.1.3 Mood outcomes...25
7.1.4 Anxiety outcomes………………………………................................25
7.1.5 Suicidality outcomes. ………………………………………………..26
7.1.6 other outcomes……………………………………………………….27
7.2 Adverse events.. 28
7.3 Evaluations by visit (table).. 28
8. Statistical analysis...29
8.1 Sample size calculation 29
8.2. General considerations... 30
8.3 Analysis of the primary outcome...30
8.4 Analysis of the secondary outcomes..31
8.5 Subgroup analysis.. 31
9. Qualitative analysis (study amendment version 2.0)………………………………….31
10. Ethics, data handling, and regulatory obligations..32
11. Steering committee..34
12. References 35

Version history and summary of changes

Version	Version date	Section	Change	Summary (changes are noted in the text)
1.0	20-11-2020	All pages	Addition	Document creation
2.0	10-01-2021		Addition	Original wording:<br>As exploratory objectives, we aim to evaluate the effect of music on patient’s treatment experiences, including the phenomenology of the treatments, operationalized using standardizing questionnaires.<br><br>New wording:<br>As exploratory objectives, we aim to evaluate the effect of music on patient’s treatment experiences, including the phenomenology of the treatments, operationalized using standardizing questionnaires and post-treatment qualitative interviews.<br><br>Rationale:<br>There is significant debate about whether subjective effects of treatment by ketamine are necessary for short and long-term benefits. Therefore, in addition to tolerability, improving participants’ subjective experiences may also increase treatment efficacity. ketamine’s subjective effects are often measured by standardized scales that do not fully capture the diversity of experiences that patients have reported. Qualitative methods are likely better suited and complementary for this task, but very few studies on ketamine in the scientific literature have employed them. To complement quantitative analysis, we propose the present study addition comparing the subjective experiences of participants undergoing ketamine treatment for treatment-resistant depression, with and without music.
3.0	30-05-2021		Deletion	Original wording:<br>Other explanatory objectives include the acute and global effect of ketamine and music on inflammatory markers before and after the first and last treatment<br>[Time Frame: Pre and Post Treatment 1 and Pre and Post treatment 6], including C-reactive protein, tumor necrosis factor-α, tumor growth factor-β1, interleukin-1β, interleukin-6, and interferon-γ<br><br>New wording:<br>Other explanatory objectives include the global effect of ketamine and music on inflammatory markers before the first and after the last treatment [Time Frame: Pre-Treatment 1 and Post treatment 6], including C-reactive protein, tumor necrosis factor-α, tumor growth factor-β1, interleukin-1β, interleukin-6, and interferon-γ.<br><br>Rationale:<br>Increased inflammatory activation of the immune system affecting both the periphery and the central nervous system is thought to play a role in the development of depression and fatigue. We aimed to explore whether

				repetitive administration of ketamine resulted in an anti-inflammatory response in treatment-resistant-depression, and whether this response was a mediator of the therapeutic effect of ketamine.<br><br>The initial aim was to explore ketamine's potential anti-inflammatory effect by measuring serum inflammatory marker levels before and immediately after the first infusion, as well as before and after the 6th infusion. We aimed to assess whether there was an acute anti-inflammatory effect in the first place, and, if present, whether it changed over the course of treatment. The second aim was to assess the overall anti-inflammatory response of repetitive ketamine infusions and to compare serum levels of the same inflammatory markers at the beginning (pre-infusion 1) and end of treatment (post-infusion 6). Because of the technical difficulty of collecting, processing, and storing biological samples, in addition to the reported discomfort of patients of having blood drawn repeatedly, the decision was made to explore only the overall anti-inflammatory response before and after the repeat treatment course of Ketamine.
4.0	08-07-2021		Deletion	Original wording:<br>Other exploratory objectives include the global effect of ketamine and music on inflammatory markers before the first and after the last treatment [Time Frame: Pre-Treatment 1 and Post treatment 6], including interleukin-6, interleukin-1β, interleukin-1ra, interleukin-10, C-reactive protein<br><br>New wording:<br>Exploratory objectives deleted<br><br>Rationale:<br>New scientific data has been published on the anti-inflammatory response of ketamine on much larger samples than ours, showing a very large variability of results. In addition, participants continued to express discomfort with having blood drawn immediately after the end of their infusion, reporting that they were not in an appropriate mental state post treatment for this type of intervention. Given tolerability issues and recently published articles, treating 30 patients (meaning 180 treatments) would have been underpowered to detect meaningful changes in inflammatory markers, the decision was made to abandon this exploratory objective.
5.0	04-08-2022		Addition	Original wording:<br>Prospective, single-center, single-blinded, randomized controlled trial with a parallel two-arm group. The study will be a single-center study performed in the Montreal area; patients will be prospectively recruited by solicitation of clinicians and by screening of ongoing referrals at the ketamine service of the Douglas Mental Health University Institute by research assistants not actively involved in their care. The existing clinic was established more than two years ago and actively receives referrals from across the province of Quebec.<br><br>New wording:<br>Prospective, two-center, single-blinded, randomized

				controlled trial with a parallel two-arm group. The study will be a two-center study performed in the Montreal area; patients will be prospectively recruited by solicitation of clinicians and by screening of ongoing referrals at the ketamine services of two academic hospitals (the Douglas Mental Health University Institute and the Jewish General Hospital) by research assistants not actively involved in their care. The existing clinics were established more than two years and 6 months ago (respectively) and actively receives referrals from across the province of Quebec.<br><br>Rationale:<br>A second ketamine service was developed in the McGill hospital network at the Jewish General Hospital following the start of the study. The service was developed by the same clinical team that established the ketamine service of the Douglas Mental Health University Institute, with an identical protocol and procedure of care. This provided an opportunity to open another study site, accelerating recruitment and study completion, and increasing external validity.

PROTOCOL SUMMARY

**Title**	**Music as an Intervention to Improve Hemodynamic Tolerability of Ketamine in Depression**
**Objectives**	Primary: To assess whether music improves hemodynamic tolerability by mitigating systolic blood pressure elevation during a course of intravenous ketamine for treatment-resistant depression<br><br>Secondary: To assess whether music improves mood, anxiety, suicidality, and psychological/physical pain during a course of intravenous ketamine for treatment-resistant depression.<br><br>Exploratory: To explore both quantitatively and qualitatively how various aspects of the treatment experience, with and without music, influence secondary outcomes.
**Study design**	Prospective, two-center, single-blinded, randomized controlled trial with a parallel two-arm group.
**Primary outcome measure**	Changes in Systolic Blood Pressure from 0 to 40 minutes of each infusion
**Secondary outcome measures**	Changes in measures of depression, anxiety, sleep, psychological and physical pain
**Population**	30 patients with Treatment resistant depression recruited and randomized into curated music (n=15) or

	control (no music) (n=15) groups
**Summary of Subjects**	Adult (18-70 years old) patients suffering from treatment-resistant depression, either unipolar or bipolar.
**Eligibility Criteria**	Patient accepted for treatment by intravenous ketamine for a current episode of treatment-resistant unipolar or bipolar depression as diagnosed by a trained psychiatrist by the following: failure to respond to at least two adequate trials of psychotropics with Level 1 evidence against bipolar and unipolar depression according to Canadian national depression guidelines.
**Screening and randomization**	After consenting, the screening will include standardized medical and psychiatric evaluation, including laboratory testing and electrocardiogram. After fulfilling all eligibility criteria, subjects will be randomized 1:1 to receive curated music or no music during their course of six IV ketamine treatments
**Description of intervention**	Music will be provided (simultaneously played on headphones for participants and on speakers in the treatment room) during all six 40-minute infusions of 0.5mg/kg ketamine administered over four weeks (biweekly for 2 weeks, then weekly for 2 weeks), beginning at the commencement of each infusion and ending 55 minutes later.
**Control Group**	Patient's six 40-minute infusions of 0.5mg/kg ketamine administered over four weeks (biweekly for 2 weeks, then weekly for 2 weeks) will be administered as per usual care, without music provided or permitted. Contact time with clinicians before, during, and after

	ketamine treatments will be matched, including accompaniment during treatments.
**Study Duration**	The study comprises a 4-week intervention (biweekly infusions for 2 weeks, then weekly for 2 weeks) with 8-week follow-up.
**Safety**	Adverse events will be summarized for each group. Adverse effects during/after ketamine infusions will be recorded.
**Funding Sources**	This work is funded by Quebec Government through le Réseau québécois sur le suicide, les troubles de l'humeur et les troubles associés.

1. Background and Rationale

Depression is one of the leading causes of morbidity and mortality worldwide, carrying massive economic, societal, and personal burdens.[1] Treatment-resistance is a growing phenomenon, and an estimated one in three of all depressed patients will fail to respond to multiple trials of available psychotherapeutic and pharmacologic treatments.[2] Bipolar depression, in particular, is considered to be especially difficult to treat.[3]

Intravenous (IV) infusions of subanesthetic doses of ketamine (most commonly 0.5mg per kg bodyweight over 40 minutes) have shown significant promise for a range of psychiatric conditions, including treatment-resistant and bipolar depression – even in cases where multiple treatments including electroconvulsive therapy have failed.[4,5] These benefits typically appear rapidly in responders, within hours or days of initial treatment, though may not persist.[4]

Ketamine infusions are typically well-tolerated and exceedingly safe when employed with appropriate precautions.[6,7] Nevertheless, ketamine is well-known as a dissociative anesthetic in that it engenders a temporary altered state of consciousness, which may be distressing. These so-called dissociative effects are amongst the leading causes of discontinuing treatment with ketamine.[7] The other major short-term transient adverse effect is increase in both blood pressure and heart rate.[5,8]

Indeed, it has been reported that blood pressures exceeding 180/100 mmHg and/or heart rate ≥110 beats per minute affect up to 30% of Treatment-Resistant Depression (TRD) patients receiving ketamine,[5] and 80% of blood pressure peak elevations during ketamine infusions (0.5 mg/kg

infused over 40 minutes) occur between 30- 40 minutes after infusion onset,[8] with a peak ketamine plasma concentration occurring at 40 minutes.[9]

One potential non-pharmacologic approach to increasing the tolerability of ketamine infusions is simultaneous music, which is anecdotally used widely in clinics worldwide and appears to be valuable in our own clinical experience.[10]Music-therapy is an evidenced-based treatment of various psychiatric disorders, and music has also been shown to be a safe and effective way of improving the tolerability of various medical procedures,[11,12] even highly invasive surgeries.[13] Numerous studies have shown that adding appropriate music to medical procedures can yield favourable hemodynamic changes,[12,14,15] improve psychological well-being,[11,14,15] and reduce inflammatory responses.[16] These benefits have been demonstrated even in critically ill patients with severely impaired consciousness in ICU settings,[11] suggesting that benefits may not be contingent on conscious appreciation.

To date, although there is reason to suspect several synergies, no study has rigorously examined the potential role of adjunctive music in subanesthetic ketamine treatments for mood disorders. Music may increase the physiological and subjective tolerance of ketamine, knowing that music has successfully counteracted hemodynamic changes in several medical procedures.[11,12] Improving the subjective experiences may also increase treatment efficacity, in addition to tolerability, as some evidence has suggested.[17]

2. Study Objectives

2.1 Primary objective

The primary objective of this study is to determine if music can mitigate systolic blood pressure increases during sub-anesthetic IV infusions of ketamine in bipolar and unipolar depressed patients receiving a course of six infusions. The central hypothesis of this trial is that music will significantly reduce the systolic blood pressure increase, thereby improving the tolerability of sub-anesthetic infusions of ketamine in the treatment of depression.

2.2 Secondary objectives

As secondary objectives, we aim to assess if music can modulate diastolic blood pressure and clinical response to repetitive sub-anesthetic IV infusions of ketamine in depression, anxiety, suicidality, sleep impairment, and psychological/physical pain.

2.3 Exploratory objectives

As exploratory objectives, we aim to evaluate the effect of music on patient's treatment experiences, including the phenomenology of the treatments, operationalized using standardizing questionnaires and post-treatment qualitative interviews.

3. Study Design

This is a prospective, two-center, single-blinded, pragmatic randomized controlled trial with a parallel two-arm design to be performed in Montreal, Canada. Patients with TRD will be recruited. After completing all screening / evaluations and baseline procedures, patients will be randomized in a 1:1 ratio to receive a curated music intervention (described below), which will consist of 60 minutes of music initiated at the beginning of each infusion (intervention group) or usual care (no music; control group) during a course of six sub-anesthetic ketamine infusions. Patients who meet all inclusion criteria and none of the exclusion criteria will be entered into the study. Recruitment criteria will be intentionally permissive in order to generate results of high external validity by including clinically representative patients who may often be excluded from clinical trials.[18] Thirty (n=30) patients are planned for enrollment in this pilot RCT. Hemodynamic evaluations will be taken at each infusion and, for the primary outcome of blood pressure, an average of the triplicate blood pressure measures by a calibrated Welch Allyn Blood Pressure Device at 0 minutes and at 40 minutes (end of infusion) will be used. Secondary outcomes will be measured at baseline and at each treatment session, as well as at 8-week follow-up. The total duration of patient participation is expected to be eight weeks including four weeks of active treatment. The total duration of the study is expected to be two years.

The study will be a two-center study performed in the Montreal area; patients will be prospectively recruited by solicitation of clinicians and by screening of ongoing referrals at the ketamine services of two academic hospitals (the Douglas Mental Health University Institute and the Jewish General Hospital) by research assistants not actively involved in their care. The

existing clinics were established more than two years and 6 months ago (respectively) and actively receives referrals from across the province of Quebec. Both clinics have successfully treated highly refractory unipolar and bipolar depressed patients. Development of those clinics included the creation and refinement of standardized assessment and treatment notes which are already integrated at both sites. An electronic medical record system capable of compiling a wide variety of clinical markers is implemented at both sites. Before study initiation and enrollment, all investigators, clinicians, and health professionals will complete study- specific training for the topic and the study protocol.

4. Study Population

Patients with a diagnosis of TRD who are accepted for treatment by IV ketamine for depression who satisfy the additional inclusion and exclusion criteria of the study will be invited to enroll. Full inclusion and exclusion criteria are provided below, in Section 4.1, where the additional criteria specific to the study (and not applicable to the ketamine clinical service) are underlined.

Patients will initially be asked if they would accept to be approached to discuss this study by a clinician. Those who agree will be contacted by an appropriately trained and certified research assistant (without clinical duties) who will present the details of the protocol and seek informed consent. If this latter consent process takes place virtually due to the COVID-19 pandemic, consenting patients will again be asked to consent in person by writing on the day of their first ketamine treatment. Before each subsequent treatment, informed consent will again be sought, thus reinforcing each patient's right to withdraw from this research protocol without influence on their care.

4.1 Inclusion Criteria

• Provision of written informed consent after reading the patient information handout.

• No changes to medications during treatment.

• Bipolar and unipolar depressive episode, current episode of depression (DSM-V) despite at least two adequate trials of psychotropics with Level 1 evidence against bipolar and unipolar depression,[19,20] as per the MADRS ≥ 20;

• Age > 18, < 75 years old.

• No active substance use disorder (beyond nicotine use disorder)

• No contraindication of ketamine

• Abstention from consuming grapefruit juice (a potent 3A4 cytochrome inhibitor) on the day of the ketamine infusions as it may alter the metabolism of ketamine.

• Ongoing psychotherapy follow-up during treatment period.

• Abstention from driving or operating heavy machinery following each ketamine infusion until either a restful night of sleep or 24 hours has elapsed.

4.2 Exclusion Criteria

- Significant hearing impairment not improved with hearing aids and/or sound amplification or unwillingness to listen to music during treatment.
- The subject's depressive symptoms have previously demonstrated non-response to esketamine or ketamine in the current major depressive episode.
- Known intellectual deficiency
- Unable to accommodate regular visits to the Depressive Disorders Program at the Douglas Mental Health University Institute or the Jewish General Hospital, Montreal, QC.
- Depression evaluated as secondary to stroke, cancer or other severe medical illnesses.
- Known risk factors for intracranial hemorrhage, including previous significant trauma, known aneurysm, or previous neurosurgery.
- Evidence of clinically relevant disease, e.g., uncontrolled hypertension, renal or hepatic impairment, significant coronary artery disease (myocardial infarct within a year prior to initial randomization), cerebrovascular disease, viral hepatitis B or C, acquired immunodeficiency syndrome.

- Prior or current substance abuse or dependence (except for caffeine or nicotine dependence) and/or recent history (last 12 months) of alcohol or cannabis abuse or dependence, as defined by DSM-5 criteria. (Cannabis will be considered similarly to alcohol for the purpose of this study, as it is clinically, in the context of its legalization. That is, recreational use that does not meet criteria for a substance use disorder and/or is not deemed to be negatively impacting patients' physical and mental health will not justify exclusion from the study just as it does not justify exclusion from purely clinical treatment by ketamine.)
- A positive toxicology screen for drugs that are not prescribed.
- Unwilling or unable to hold benzodiazepines from the evening prior to the infusion of ketamine.
- Unwilling or unable to discontinue any narcotic beginning a minimum of 5 drug half-lives prior to infusion.
- Unwilling or unable to discontinue memantine (an NMDA antagonist) during infusions, beginning a minimum of 5 drug half-lives prior to infusions.
- Pregnant, lactating, or of childbearing potential and not willing to use an approved method of contraception during the ketamine infusion, as per above.
- A clinical finding that is unstable or that, in the opinion of the treating clinician(s), would be negatively affected by, or would affect, the medication (e.g., diabetes mellitus, unstable angina).
- Liver function tests AST and ALT three times the upper normal limit at screening.
- ECG results considered significantly abnormal as determined by the clinician(s).
- History of seizure disorder, except febrile convulsions.
- Known history of intolerance or hypersensitivity to ketamine.
- Acute psychotic symptoms, as judged by the initial clinical interview or reported by referring clinicians.

- Any significant, recent, acute decline in exercise tolerance.
- Uncorrected hypothyroidism or hyperthyroidism.
    - Subjects needing a thyroid hormone supplement to treat hypothyroidism must have been on a stable dose of the medication for 3 months prior to beginning infusions.
    - Clinically significant deviation from the reference range in clinical laboratory test results as judged by the clinician(s).

4.3 Psychotropics and antihypertensive medications

All patients should be maintained on the same psychotropics and antihypertensive medications throughout the treatment month, as medically feasible, with no introduction of new therapies. Changes in current medication and/or administration of new medication will be registered; changes in psychotropics and antihypertensive medication influencing directly or indirectly blood pressure control or mood / anxiety / suicidality / sleep symptoms will be registered.

4.4 Randomization Process

Patients who successfully complete the screening assessments will be randomized into the study. Eligible patients will be 1:1 randomized to the music-intervention (intervention, n=15) or usual care (control, n=15) groups. Participants will be randomized by an independent third party affiliated with McGill University using a computer-generated randomized sequence. The randomization sequence code will stored securely for the duration of the trial. The only theoretical exception for the code to be broken will be any serious adverse event and need for subsequent participant withdrawal.

4.5 Screen failures and participant withdrawal or discontinuation from study

Patients who are ineligible for the study based on screening assessments or decide to discontinue the study will be registered as such. Reason(s) for failing screening or discontinuing will be recorder.

5. Study endpoints

5.1 Primary outcome measure

Change in systolic blood pressure (SBP) at 40 minutes (end of infusion) versus 0 minutes (baseline) between intervention and control groups.

5.2 Secondary outcome measures (study amendment version 2.0)

- Change in diastolic blood pressure (DBP) at 40 minutes versus at 0 minutes between intervention and control group.
- Change from baseline in mood symptoms to last treatment [Time Frame: Baseline, Week 4]
- Change from baseline in mood symptoms to Follow-up [Time Frame: Baseline, Week 8]
- Change from baseline in anxiety symptoms to last treatment [Time Frame: Baseline, Week 4]
- Change from baseline in anxiety symptoms to Follow-up [Time Frame: Baseline, Week 8]
- Change from baseline in suicidality symptoms to last treatment [Time Frame: Baseline, Week 4]
- Change from baseline in suicidality symptoms to Follow-up [Time Frame: Baseline, Week 8]
- Change from baseline in sleep symptoms to last treatment [Time Frame: Baseline, Week 4]
- Change from baseline in sleep symptoms to Follow-up [Time Frame: Baseline, Week 8]
- Change from baseline in Psychological and Physical Pain to last treatment [Time Frame: Baseline, Week 4]
- Change from baseline in Psychological and Physical Pain to Follow-up [Time Frame: Baseline, Week 8]

- Experiential questionnaires administered at every session immediately following infusion
- Subjective experiences of patients undergoing ketamine therapy assessed by semi-structured interviews and audio recording [Time Frame: Week 5 to 6]

6. Study intervention and control groups

The ketamine treatment procedure received by both the intervention and control groups will consist of six infusions given over four weeks. During the first two weeks, infusions will be given bi-weekly (on Mondays and Wednesdays) and weekly during the last two weeks (on Wednesdays). The patients will receive their infusions in a quiet room, in a semi-reclined position on a hospital bed or medical recliner at an angle of approximately 45 degrees. A vein will be cannulated and 0.5mg/kg of ketamine will be diluted in 250mL of normal saline by the treating team's nurse, according to clinical information (i.e. weight, height and BMI) and verified by one other member of the treating team before being administered continuously during 40-minute infusions. In patients with a BMI greater than 30, ketamine doses will be calculated based on the upper-limit BMI of 30, given that greater hemodynamic changes with a body mass index of 30 and above have been observed.[7]

After cannulation and setup of the infusion equipment, patients will be left alone for ten minutes in order to allow an accurate measurement of baseline blood pressure. For these ten minutes, they will be asked to remain in the same semi-reclined position, in silence and without use of electronic devices such as cell phones. Clinicians will then return to the room and measure blood pressure in triplicate before beginning the infusion. The ketamine infusions will be given in the presence of the nurse and a physician with ongoing assessments of patients' physiological and mental status during the infusion. All patients will be offered blindfolds for the duration of the infusions.

6.1 Study intervention: Music during ketamine treatment

The intervention consists of playing music via headphones during all ketamine treatments, beginning at the commencement of each infusion and ending approximately 60 minutes later. For

the purposes of this study, several music playlists have been created based on scientific literature regarding the use of music with hallucinogen-induced psychoactive drugs. [21]Specifically, the playlists are composed of various pieces of music of diverse genres mostly taken from published playlists used in psilocybin-assisted psychotherapy.[22] The music used in the playlist is free from understandable lyrics; i.e., no human vocals in a language that the patient understands. The specific songs and their order were chosen to create playlists that differ in style but are cohesive and follow a similar pattern: calming at the outset, then richer and more absorbing after approximately 10-15 minutes, then again calming for the last 20 minutes of the infusion (beginning around 35 minutes; five minutes before the 40-minute infusion endpoint). The final playlists used in the study will be published alongside the trial's findings.

On the day of each infusion, before the treatment begins, clinicians will discuss music choices with participants to select amongst one of the available playlists based on their preferences, mood, and experiences with other playlists (for subsequent treatments). This may entail briefly listening to excerpts of the selected playlists. Generally, patients will asked to select unfamiliar music in accordance with recommendation from the psilocybin literature. [21]After the first treatment, patients will be invited to request the incorporation of music of their choosing into the middle part of the playlists (i.e., ending no later than five minutes before the infusion termination). If requested, service clinicians will modify one of the study playlists to include the patient's preferred song(s). Following the active treatment period, patients will be sent a copy of the playlists used in their treatments.

Compliance with the music intervention will be assessed in a binary fashion (yes / no) for each session depending on if the patient listened to the music playlist throughout the ketamine infusion (from 0 minute to 55 minutes). The treating nurse in the room will be asked to keep a log noting compliance for every session.

6.2 Control condition: treatment as usual

The control condition consists of receiving ketamine in the same settings, setup in the same way (except for any music listening equipment), with blindfolds, and with matched accompaniment relative to the intervention arm. The only difference between conditions is the use of music. As needed, participants in the control condition will be provided with reassurance and basic breathing or mindfulness exercises by their clinicians to encourage relaxation during the infusions.

The amount of contact with clinicians will also be matched outside of the ketamine treatments. In lieu of choosing music collaboratively with their clinician at study visits before the ketamine infusion, patients will have equivalent amount of contact with their clinicians. This time is not specifically structured but will entail further discussion of personal or psychiatric issues, responses to the treatment process thus far (for subsequent treatments), and relaxation exercises.

7. Study procedures and guidelines

Written informed consent will be obtained before performing any procedure. The principal investigators are responsible for ensuring that all assessments are performed according to the protocol and that the appropriate data are recorded in the appropriate forms.

7.1 Clinical assessments

7.1.1 Demographics, medical history, and medication

All patients will undergo a 60- to 120-minute psychiatric and medical evaluation with one of physician investigators, including the reviewing of laboratory and electrocardiogram test results, to determine their eligibility for treatment by intravenous ketamine. Accepted patients will then be scheduled to meet with the clinic's physicians to collaboratively develop a biopsychosocial

treatment plan and to arrange for concomitant psychological support (one hour of evidence-based psychotherapy with any confirmed clinician) during the treatment course. The broad aim of these plans is to optimize the chances for significant benefits with the course of ketamine, and to decrease the odds of subsequent depression relapse. Demographic information, relevant clinical information, including history of current disease, will be recorded. All current and past medication and/or complementary therapies, including psychotherapies and neurostimulation, will be documented. All patients will have 1-2 additional 30-minute appointments with the clinicians before their initial ketamine treatment to further discuss the ketamine treatments, address any questions/concerns, and to reinforce psychosocial treatment plans.

7.1.2 Blood pressure and hemodynamic function

Prior to every infusion and at 15, 30 and 40 minutes (end of infusion), baseline vital signs, including heart rate, respiratory rate, oxygen saturation, and blood pressure will be measured. Blood pressure change measurement will be assessed by a validated and calibrated Welch Allyn Blood Pressure Device according to Hypertension Canada Guidelines and recommended technique for automated office blood pressure.[23] The patient will be semi-reclined (approximately 45 degrees) on the treatment bed or medical lounger, the back supported, the arm bare and supported, legs uncrossed, not talking or moving before or during the measurement, using a cuff size appropriate for the arm (i.e bladder width close to 40% of the arm circumference and length cover 80-100% of the arm circumference), the middle of the cuff at heart level, with the lower edge of the cuff 3 cm above the elbow crease. The initial measurement will be taken after 10 minutes of quiet rest, as described above. No change in bed/recliner position will be permitted during or between ketamine infusions. This technique will be used to measure the change in systolic blood pressure (SBP) at 40 minutes versus at 0 minutes between intervention and control groups. Specifically, we will treat the difference between the average of the triplicate SBP measurements at 0 minutes (measurement taken at 1 minute interval as recommended) and the

average of the triplicate SBP measurements at 40 minutes (measurement taken at 1 minute interval), at each infusion.

7.1.3 Mood outcomes

Mood related outcomes will be evaluated by different self-report questionnaires and clinician-rated scale as detailed below (before the infusions at treatment visits). A trained clinician such as a psychiatrist or a research assistant with at least a master's degree, blinded to the patient assignment, will complete the clinician-rated questionnaires.

1. Change From Baseline in Montgomery-Asberg depression rating scale (MADRS) Total Score to Last Treatment [Time Frame: Baseline, Week 4] [24]
2. Change From Baseline in MADRS Total Score to Follow-up [Time Frame: Baseline, 1-month follow-up (8 weeks)] [24]
3. Change From Baseline in Beck Depression Inventory (BDI-II) Total Score to Last Treatment [Time Frame: Baseline, Week 4] [25]
4. Change From Baseline in Beck Depression Inventory (BDI-II) Total Score to Follow-up [Time Frame: Baseline, 1-month follow-up (8 weeks)] [25]
5. Change From Baseline in Clinician-Rated Global Impression Improvement Score to Last Treatment [Time Frame: Baseline, Week 4] [26]
6. Change From Baseline in Clinician-Rated Global Impression Severity Score to Last Treatment [Time Frame: Baseline, 1-month follow-up (8 weeks)] [26]

7.1.4 Anxiety outcomes

Anxiety related outcomes will be evaluated by self-report questionnaires as detailed below (before the infusions at treatment visits).

1. Change From Baseline in STAI-Y A to Last Treatment [Time Frame: Baseline, Week 4] [27]

2. Change From Baseline in STAI-Y A to Follow-up [Time Frame: Baseline, 1-month follow-up (8 weeks)] [27]
3. Change From Baseline in STAI-Y B to Last Treatment [Time Frame: Baseline, 1-month follow-up (8 weeks)] [27]
4. Change From Baseline in STAI-Y B to Follow-up [Time Frame: Baseline, 1-month follow-up (8 weeks)] [27]

7.1.5 Suicidality outcomes

Suicidality-related outcomes will be evaluated by different self-report questionnaires and clinician-rated scale as detailed below (before the infusions at treatment visits). A trained clinician such as a psychiatrist or a research assistant with at least a master's degree, blinded to the patient assignment, will complete the clinician-rated questionnaires.

1. Change From Baseline in Beck Scale for Suicide Ideation (SSI) Total to Last Treatment [Time Frame: Baseline, Week 4] [28]
2. Change From Baseline in Beck Scale for Suicide Ideation (SSI) Total to Follow-up [Time Frame: Baseline, 1-month follow-up (8 weeks)] [28]
3. Change From Baseline in Clinician-Rated Global Impression Suicidality Severity Score to Last Treatment [Time Frame: Baseline, Week 4] [26]
4. Change From Baseline in Clinician-Rated Global Impression Suicidality Improvement Score to Last Treatment [Time Frame: Baseline, Week 4] [26]
5. Change From Baseline in Clinician-Rated Global Impression Suicidality Severity Score to Follow-up [Time Frame: Baseline, 1-month follow-up (8 weeks)] [26]
6. Change From Baseline in Clinician-Rated Global Impression Suicidality Improvement Score to Follow-up [Time Frame: Baseline, 1-month follow-up (8 weeks)] [26]

7.1.6 Other

Psychological and physical pain, sleep and experience-related outcomes will be evaluated by different self-reported questionnaire as detailed below (before the infusions at treatment visits unless otherwise specified). Qualitative assessment of subjective experiences of patients undergoing ketamine therapy will be performed by semi-structured interviews lasting 1 to 2 hours and scheduled one or two weeks after all the dosing sessions. In addition, patient-clinician interactions, including the dosing period and the debriefing, will be recorded for each session.

1. Sleep: Change From Baseline in PSQI Total and Subscores to Last Treatment
   [Time Frame: Baseline, Week 4] [29]
2. Sleep: Change From Baseline in PSQI Total and Subscores to Follow-up
   [Time Frame: Baseline, 1-month follow-up (8 weeks)] [29]
3. Change From Baseline in Psychological and Physical Pain to Last Treatment
   [Time Frame: Baseline, Week 4] [30]
4. Change From Baseline in Psychological and Physical Pain to Follow-up
   [Time Frame: Baseline, 1-month follow-up (8 weeks)] [30]
5. The Mystical Experience Questionnaire (MEQ) after each treatment
   [Time Frame: Immediately after the interventions] [31]
6. The Emotional Breakthrough Inventory (EBI) after each treatment
   [Time Frame: Immediately after the interventions] [32]
7. The Psychedelic Music Questionnaire Short-Form (PMQ-SF) after each treatment
   [Time Frame: Immediately after the interventions]
8. Qualitative assessment using interpretative phenomenological analysis (IPA) of patients subjective experiences based on semi-structured interviews conducted 1 to 2 week after completion of all dosing sessions, and by audio recordings of patient-clinician interactions during every dosing session

7.2 Adverse events

Information regarding the occurrence of potential adverse effects during/after ketamine sessions (e.g. headache, nausea and vomiting, neuropsychiatric symptoms, hypertensive peak, etc.) will be captured throughout the study in both groups.

7.3 Evaluations by visit

The following table lists the assessment procedures and indicates (X) in which visits they are performed. Written informed consent will be obtained before performing any procedure. The sites investigators are responsible for ensuring that all assessments are performed according to the protocol, and that data are recorded in the appropriate forms. Missed or partial visits/ assessments must be reported on the case report forms (reasons should be provided).

	**Phase**			
**Data collection**	**Screening & Preparation**	**Pre-infusion**	**Post infusion**	**Follow-up**
Demographics	x			
Medical and Psychiatric Hx	x			
Medication	x			
Physical examination and BMI	x			
Informed consent form	x			
Inclusion / exclusion criteria	x			
Blood Pressure	x	x	x	
Mood questionnaire (self-reported)		x		x
Mood questionnaire (clinician-rated)		x		x

Anxiety questionnaires (self-reported)		x		x
Suicidality questionnaires (self-reported)		x		x
Suicidality questionnaires (clinician-rated)		x		x
Sleep questionnaire (self reported)		x		x
Psychological & Physical Pain questionnaires		x		x
Experiential questionnaires			x	
Semi-structured interviews				x

8. Statistical analysis

8.1 Sample size calculation

This study is a pilot trial. There is currently limited data to justify the sample size, given the innovative nature of studying music on hemodynamic tolerance during sub-anesthetic ketamine infusions. Furthermore, performing a priori power analyses with a within-subject factor is complicated and difficult to perform accurately without a prior study to justify correlations between levels and sphericity assumptions.[33-35] This pilot RCT will thus provide the necessary and adequate data to plan a subsequent confirmatory RCT.[34]

A preliminary power analysis was done to estimate the number of patients required for this study and computed a priori using the software G*Power 3.1, University Düsseldorf, Germany.[36] The sample size calculation was based on the results of Mok et al. who studied the impact of music on hemodynamic functioning in preoperative patients, given the similar within-between study design and procedure.[37] They found pre-post differences of systolic blood pressure of -3,5 ± 14.5 mmHg in the music group and no changes (+0,1 ± 10,3 mmHg) in the control group.[37] A two-sided 0.05

level of significance and a sample size of 28 patients (14 patients per group), each providing six pre-post datapoints, provided 80% statistical power to significantly demonstrate this difference in systolic blood pressure, assuming sphericity of 1 and correlations among measures of 0.5. To accommodate for a 10% attrition rate (given low attrition rate in ketamine trials for depression, [7] we will recruit a total of 30 patients (G*power 3.1, ANOVA Repeated measures, within between interaction), a feasible recruitment rate at the current clinical capacities over 12-18 months.

8.2. General considerations

The analyses will be performed based on the intention- to-treat principle.[18] Baseline variables by treatment groups will be characterized in a summary table. Between-group differences at baseline will be inspected and judged according to clinical relevance. The assessment of compliance with the intervention and safety/tolerability will be summarized for both groups. Adverse events, if any, will be reported by treatment group.

8.3 Analysis of the primary outcome

For the primary outcome data analysis, we will adopt the generalized linear model (GLM) to investigate the change in systolic blood pressure (SBP) at 40 minutes versus at 0 minutes between intervention and control groups.[38] Specifically, we will treat the difference between the average of the triplicate SBP measurements at 0 minutes and 40 minutes at each infusion as the outcomes in the GLM, adjusting for covariates such as intervention, age and sex. The generalized estimating equation (GEE) technique is proposed to estimate the regression coefficients, and the corresponding variances are estimated by the sandwich estimators.[39] GEE will be used to account for the clustering (non-independence) of the data attributed to the longitudinal and repeated measures design of this study.[39] A Gaussian regression with an identity link for continuous outcomes will be used to estimate mean differences between treatment groups, fitted with robust error estimation and an ar1 working correlation structure. We will use Inverse-probability

weighting (IPW) to account for missing data and augmentation (AUG) for imbalance in covariates between treatment groups for doubly robust GEE estimation.[40]

8.4 Analysis of the secondary outcomes

The change from baseline to the end of the treatment month and to the 8-week follow-up end-point will be analyzed using traditional end-point analysis (paired T-test) and latent growth curve analysis.

8.5 Subgroup analysis

There are no planned subgroup analyses given the relatively small sample size in the context of repeated measure study design.

9. Qualitative analysis

To investigate subjective experiences of patients undergoing ketamine therapy, we will utilize qualitative methods, more specifically semi-structured interviews and audio recordings of patient-clinician interactions. This will provide a rich multifaceted description of phenomena and bring about a fuller understanding complementing the breadth of understanding provided by quantitative methods. This additional effort will consequently help us interpret and contextualize the quantitative results to better understand the potential role of adjunctive music in subanesthetic ketamine treatments for mood disorders and its associated dissociative effects, which will, moreover, validate the quantitative data being collected in parallel.

Interpretative phenomenological analysis (IPA) is a methodology often used in medical qualitative research in general, as well as in psychedelic research in particular.[41] It focusses on how people make sense of their lived experience, especially in contexts where little is known of the phenomena under study. This particular method of analysis has been used before to investigate the benefits of music therapy interventions in cancer care settings and in group music

therapy for depression,[42,43] and thus is deemed appropriate in our case to investigate the complexity of patients' subjective experience of music during the therapeutic sessions.

The research team will recruit participants (a maximum of 20) in order to conduct semi-directive interviews with members of both groups, music and no music. The interviews are planned to last between 1 and 2 hours and will be scheduled one or two weeks after all the dosing sessions. In addition, patient-clinician interactions, including the dosing period and the debriefing, will be recorded for each session. Both the interviews and the interactions from the treatment sessions will be anonymized and transcribed by a professional service

Analysis Process

Once all the data is collected, two research assistants will act as coders. They will individually read and code two transcripts, the same, from each group (two transcripts, one from the music group and one from the "no music" group). After this first step, the coders will meet with one study PI to discuss emergent patterns and themes. The team will find superordinate themes and connections between themes in order to construct a coding guide for each subsample. The team will hold an analysis journal and note the decision trail, the main discussion topics and the theoretical reflections. Coders will then alternate between coding a transcript of a participant having receiving music and no music. The team will meet after coding each subsequent transcript to discuss emerging themes and refine previous ones. After coding all transcripts, the team will do a cross-case analysis in order to identify themes specific to each subsample.

10. Ethics, data handling, and regulatory obligations

- All currently solicited and collected information that is recorded on paper as part of the clinical service, such as clinical or demographic details, will be stored in the patients' ketamine charts via the same process that currently takes place.

- Patients will be randomly assigned a unique research identifier upon enrollment. Questionnaires that are administered electronically will contain only this unique identifier (though indirect identifiers like email addresses will also be collected). As takes place in the current clinical workflow, patient identifiers for paper questionnaires are added by hand once they have been printed.
- The digital questionnaires (which contain only the research identifiers) will be stored using the commercial clinical and research software AdvancedCare (https://advancedcare.com). This software is compliant with HIPPA and all Canadian legislation and includes enterprise-grade encryption (AES-256). The data is stored only within Canada and does not leave the jurisdiction for any purposes including transit or backup. The software is used in clinical and research services across Canada using full patient details including electronic patient records. As an added layer of security, we will store no direct identifying patient information on this software.
- In order to permit data analysis, an encrypted database of results (Excel spreadsheet) containing no direct patient identifiers will be made and stored securely at each site for local patients. At the end of the study, data will be transferred to the Douglas Mental Health University Institute to assemble the full database of results according to the interinstitutional Data Transfer and Sharing Agreement.
- The list of unique patient identifiers will be store only on an encrypted file on the Douglas Mental Health University Institute local servers, and will not leave the institute.
- All data that contains patient identifiers will remain in the local paper charts as described above. Only the principal investigators will have access the encrypted file linking patient identifiers to clinical data. Only these individuals and one appropriately trained research assistant will have access to identified patient data, and only in the context of building the anonymous database and/or providing clinical services.

- The full study database will only be accessible by appropriately trained members of the research team.
- Data will be stored and accessible for a maximum of 7 years after the conclusion of the study.

11. Steering committee

Given the relatively small size of the trial and the fact that patients receive treatments as part of clinical services with significant clinical and research oversight by their institutions, we do not plan to have a specific trial steering committee.

12. References

1. Weinberger AH, Gbedemah M, Martinez AM, Nash D, Galea S, Goodwin RD. Trends in depression prevalence in the USA from 2005 to 2015: widening disparities in vulnerable groups. *Psychol Med*. 2018;48(8):1308-1315.
2. Demyttenaere K, Van Duppen Z. The impact of (the concept of) treatment-resistant depression: an opinion review. *Int J Neuropsychopharmcol*. 2019;22(2):85-92.
3. Geddes JR, Miklowitz DJ. Treatment of bipolar disorder. *The lancet*. 2013;381(9878):1672-1682.
4. Alnefeesi Y, Chen-Li D, Krane E, et al. Real-world effectiveness of ketamine in treatment-resistant depression: A systematic review & meta-analysis. *J Psychiatr Res*. Jul 2022;151:693-709. doi:10.1016/j.jpsychires.2022.04.037
5. McIntyre RS, Rosenblat JD, Nemeroff CB, et al. Synthesizing the Evidence for Ketamine and Esketamine in Treatment-Resistant Depression: An International Expert Opinion on the Available Evidence and Implementation. *Am J Psychiatry*. May 1 2021;178(5):383-399. doi:10.1176/appi.ajp.2020.20081251
6. Rodrigues NB, McIntyre RS, Lipsitz O, et al. Safety and tolerability of IV ketamine in adults with major depressive or bipolar disorder: results from the Canadian rapid treatment center of excellence. *Expert Opin Drug Saf*. 2020;19(8):1031-1040.
7. Wan LB, Levitch CF, Perez AM, et al. Ketamine safety and tolerability in clinical trials for treatment-resistant depression. *J Clin Psychiatry*. Mar 2015;76(3):247-52. doi:10.4088/JCP.13m08852
8. Riva-Posse P, Reiff CM, Edwards JA, et al. Blood pressure safety of subanesthetic ketamine for depression: a report on 684 infusions. *J Affect Disord*. 2018;236:291-297.
9. Zanos P, Moaddel R, Morris PJ, et al. Ketamine and ketamine metabolite pharmacology: insights into therapeutic mechanisms. *Pharmacological reviews*. 2018;70(3):621-660.
10. Greenway KT, Garel N, Goyette N, Turecki G, Richard-Devantoy S. Adjunctive music improves the tolerability of intravenous ketamine for bipolar depression. *International Clinical Psychopharmacology*. 2021;36(4):218-220.
11. Bradt J, Dileo C. Music interventions for mechanically ventilated patients. *Cochrane database of systematic reviews*. 2014;(12)
12. Merakou K, Varouxi G, Barbouni A, et al. Blood pressure and heart rate alterations through music in patients undergoing cataract surgery in Greece. *Ophthalmology and eye diseases*. 2015;7:OED. S20960.
13. Byers J, Smyth K. Effect of a music intervention on noise annoyance, heart rate, and blood pressure in cardiac surgery patients. *American Journal of Critical Care*. 1997;6(3):183-191.
14. Wiwatwongwana D, Vichitvejpaisal P, Thaikruea L, Klaphajone J, Tantong A, Wiwatwongwana A. The effect of music with and without binaural beat audio on operative anxiety in patients undergoing cataract surgery: a randomized controlled trial. *Eye*. 2016;30(11):1407-1414.
15. Bradt J, Dileo C, Magill L, Teague A. Music interventions for improving psychological and physical outcomes in cancer patients. *Cochrane Database of Systematic Reviews*. 2016;(8)
16. Conrad C, Niess H, Jauch K-W, Bruns CJ, Hartl WH, Welker L. Overture for growth hormone: requiem for interleukin-6? *Crit Care Med*. 2007;35(12):2709-2713.

17. Kaelen M, Giribaldi B, Raine J, et al. The hidden therapist: evidence for a central role of music in psychedelic therapy. *Psychopharmacology (Berl)*. 2018;235:505-519.
18. Haynes RB. *Clinical epidemiology: how to do clinical practice research*. Lippincott williams & wilkins; 2012.
19. Kennedy SH, Lam RW, McIntyre RS, et al. Canadian Network for Mood and Anxiety Treatments (CANMAT) 2016 clinical guidelines for the management of adults with major depressive disorder: section 3. Pharmacological treatments. *The Canadian Journal of Psychiatry*. 2016;61(9):540-560.
20. Yatham LN, Kennedy SH, Parikh SV, et al. Canadian Network for Mood and Anxiety Treatments (CANMAT) and International Society for Bipolar Disorders (ISBD) 2018 guidelines for the management of patients with bipolar disorder. *Bipolar Disord*. 2018;20(2):97-170.
21. Johnson MW, Richards WA, Griffiths RR. Human hallucinogen research: guidelines for safety. *Journal of psychopharmacology*. 2008;22(6):603-620.
22. Davis AK, Barrett FS, May DG, et al. Effects of psilocybin-assisted therapy on major depressive disorder: a randomized clinical trial. *JAMA Psychiatry*. 2021;78(5):481-489.
23. Rabi DM, McBrien KA, Sapir-Pichhadze R, et al. Hypertension Canada's 2020 comprehensive guidelines for the prevention, diagnosis, risk assessment, and treatment of hypertension in adults and children. *Canadian Journal of Cardiology*. 2020;36(5):596-624.
24. Galinowski A, Lehert P. Structural validity of MADRS during antidepressant treatment. *International Clinical Psychopharmacology*. 1995;10(3):157-161.
25. Beck AT, Steer RA, Ball R, Ranieri W. Comparison of Beck Depression Inventories -IA and -II in psychiatric outpatients. *J Pers Assess*. Dec 1996;67(3):588-97. doi:10.1207/s15327752jpa6703_13
26. Busner J, Targum SD. The clinical global impressions scale: applying a research tool in clinical practice. *Psychiatry (edgmont)*. 2007;4(7):28.
27. Spielberger C, Gorsuch R, Lushene R, Vagg P, Jacobs G. Consulting Psychologists Press; Palo Alto, CA: 1983. *Manual for the state-trait anxiety inventory*. 1983;
28. Beck AT, Kovacs M, Weissman A. Assessment of suicidal intention: the Scale for Suicide Ideation. *J Consult Clin Psychol*. Apr 1979;47(2):343-52. doi:10.1037//0022-006x.47.2.343
29. Carpenter JS, Andrykowski MA. Psychometric evaluation of the Pittsburgh sleep quality index. *Journal of psychosomatic research*. 1998;45(1):5-13.
30. Jollant F, Voegeli G, Kordsmeier NC, et al. A visual analog scale to measure psychological and physical pain: A preliminary validation of the PPP-VAS in two independent samples of depressed patients. *Progress in Neuro-Psychopharmacology and Biological Psychiatry*. 2019;90:55-61.
31. Barrett FS, Johnson MW, Griffiths RR. Validation of the revised Mystical Experience Questionnaire in experimental sessions with psilocybin. *Journal of Psychopharmacology*. 2015;29(11):1182-1190.
32. Roseman L, Haijen E, Idialu-Ikato K, Kaelen M, Watts R, Carhart-Harris R. Emotional breakthrough and psychedelics: validation of the emotional breakthrough inventory. *Journal of psychopharmacology*. 2019;33(9):1076-1087.

33. Guo Y, Logan HL, Glueck DH, Muller KE. Selecting a sample size for studies with repeated measures. *BMC medical research methodology*. 2013;13(1):1-8.
34. Birkett MA, Day SJ. Internal pilot studies for estimating sample size. *Statistics in medicine*. 1994;13(23-24):2455-2463.
35. Cohen J. Statistical power analysis. *Current directions in psychological science*. 1992;1(3):98-101.
36. Faul F, Erdfelder E, Lang A-G, Buchner A. G* Power 3: A flexible statistical power analysis program for the social, behavioral, and biomedical sciences. *Behavior research methods*. 2007;39(2):175-191.
37. Mok E, Wong K-Y. Effects of music on patient anxiety. *AORN journal*. 2003;77(2):396-410.
38. McCullagh P. *Generalized linear models*. Routledge; 2019.
39. Burton P, Gurrin L, Sly P. Extending the simple linear regression model to account for correlated responses: an introduction to generalized estimating equations and multi-level mixed modelling. *Statistics in medicine*. 1998;17(11):1261-1291.
40. Prague M, Wang R, Stephens A, Tchetgen Tchetgen E, DeGruttola V. Accounting for interactions and complex inter-subject dependency in estimating treatment effect in cluster-randomized trials with missing outcomes. *Biometrics*. 2016;72(4):1066-1077.
41. Belser AB, Agin-Liebes G, Swift TC, et al. Patient experiences of psilocybin-assisted psychotherapy: an interpretative phenomenological analysis. *Journal of Humanistic Psychology*. 2017;57(4):354-388.
42. Windle E, Hickling LM, Jayacodi S, Carr C. The experiences of patients in the synchrony group music therapy trial for long-term depression. *The Arts in Psychotherapy*. 2020;67:101580.
43. Pothoulaki M, MacDonald R, Flowers P. An interpretative phenomenological analysis of an improvisational music therapy program for cancer patients. *Journal of Music Therapy*. 2012;49(1):45-67.
