# Supplementary material for: Music as an Intervention to Improve the Hemodynamic Response of Ketamine in Depression: A Randomized Clinical Trial
Source: JAMA Netw Open. 2024 Feb 5;7(2):e2354719. doi: 10.1001/jamanetworkopen.2023.54719 (PMC10845001; doi:10.1001/jamanetworkopen.2023.54719)
Supplement: Supplement 2. — eMethods eTable 1. Baseline Demographics and Clinical Characteristics eTable 2. Musical Playlists Employed in the Study eReferences [file jamanetwopen-e2354719-s002.pdf]

Supplemental Online Content

Greenway KT, Garel N, Dinh-Williams LL, et al. Music as an intervention to improve the hemodynamic response of ketamine in depression: a randomized clinical trial. *JAMA Netw Open*. 2024;7(2):e2354719. doi:10.1001/jamanetworkopen.2023.54719

eMethods.
eTable 1. Baseline Demographics and Clinical Characteristics
eTable 2. Musical Playlists Employed in the Study
eReferences

This supplemental material has been provided by the authors to give readers additional information about their work.

© 2024 Greenway KT et al. *JAMA Network Open.*

eMethods

Participants

Patients with a diagnosis of TRD who were accepted for treatment by IV ketamine for depression at the study sites who satisfied these additional study inclusion criteria were invited to enroll: provision of informed consent to participate in the study, agreement to maintain their current medication regimen throughout the study, and absence of significant hearing impairment. The inclusion and exclusion criteria were intentionally broad to increase the external validity of the study, in alignment with its pragmatic design. All patients additionally satisfied the inclusion and exclusion criteria of the ketamine services at the Douglas Mental Health University Institute and the Jewish General Hospital:

Inclusion criteria:

- Bipolar and unipolar depressive episode, current episode of depression (DSM-5) despite at least two adequate trials of psychotropics with Level 1 evidence against bipolar or unipolar depression,[1,2]
- Montgomery-Åsberg Depression Rating Scale (MADRS) ≥ 20;[3]
- Age > 18, < 75 years old.
- No active substance use disorder (beyond nicotine use disorder)
- Abstention from consuming grapefruit juice (a potent 3A4 cytochrome inhibitor) on the day of the ketamine infusions as it may alter the metabolism of ketamine.
- Ongoing psychotherapy follow-up during treatment period
- Abstention from driving or operating heavy machinery following each ketamine infusion until either a restful night of sleep or 24 hours has elapsed.

Exclusion criteria:

- The subject’s depressive symptoms have previously demonstrated non-response to esketamine or ketamine in the current major depressive episode.
- Known intellectual deficiency.
- Unable to accommodate regular visits to the Depressive Disorders Program at the Douglas Mental Health University Institute or the Jewish General Hospital, Montreal, QC.
- Depression evaluated as secondary to stroke, cancer or other severe medical illnesses.
- Known risk factors for intracranial hemorrhage, including previous significant trauma, known aneurysm, or previous neurosurgery.
- Evidence of clinically relevant disease, e.g., uncontrolled hypertension, renal or hepatic impairment, significant coronary artery disease (myocardial infarct within a year prior to initial randomization), cerebrovascular disease, viral hepatitis B or C, acquired immunodeficiency syndrome.
- Prior or current substance abuse or dependence (except for caffeine or nicotine dependence) and/or recent history (last 12 months) of alcohol or cannabis abuse or

© 2024 Greenway KT et al. *JAMA Network Open.*

dependence, as defined by DSM-5 criteria. (Cannabis will be considered similarly to alcohol for the purpose of this study, as it is clinically, in the context of its legalization. That is, recreational use that does not meet criteria for a substance use disorder and/or is not deemed to be negatively impacting patients' physical and mental health will not justify exclusion from the study just as it does not justify exclusion from purely clinical treatment by ketamine.)

- A positive toxicology screen for drugs that are not prescribed.
- Unwilling or unable to hold benzodiazepines from the evening prior to the infusion of ketamine.
- Unwilling or unable to discontinue any narcotic beginning a minimum of 5 drug half-lives prior to infusion.
- Unwilling or unable to discontinue memantine (an NMDA antagonist) during infusions, beginning a minimum of 5 drug half-lives prior to infusions.
- Pregnant, lactating, or of childbearing potential and not willing to use an approved method of contraception during the ketamine infusion, as per above.
- A clinical finding that is unstable or that, in the opinion of the treating clinician(s), would be negatively affected by, or would affect, the medication (e.g., diabetes mellitus, unstable angina).
- Liver function tests AST and ALT three times the upper normal limit at screening.
- ECG results considered significantly abnormal as determined by the clinician(s).
- History of seizure disorder, except febrile convulsions.
- Known history of intolerance or hypersensitivity to ketamine.
- Acute psychotic symptoms, as judged by the initial clinical interview or reported by referring clinicians.
- Any significant, recent, acute decline in exercise tolerance.
- Uncorrected hypothyroidism or hyperthyroidism.
    - Subjects needing a thyroid hormone supplement to treat hypothyroidism must have been on a stable dose of the medication for 3 months prior to beginning infusions.
    - Clinically significant deviation from the reference range in clinical laboratory test results as judged by the clinician(s).

Ketamine service screening process and study enrollment

The study was performed at the ketamine services of two academic hospitals (the Douglas Mental Health University Institute and the Jewish General Hospital). Patients are referred from psychiatrists across the province of Quebec to these ketamine services to receive treatment for highly treatment-refractory unipolar and bipolar depression.

All referred patients undergo a comprehensive medical and psychiatric evaluation, including the review of laboratory and electrocardiogram test results, to determine eligibility for intravenous ketamine treatment. Accepted patients meet with the ketamine service psychiatrist to collaboratively develop a treatment plan and arrange for psychological support. This process typically takes 120 minutes.

© 2024 Greenway KT et al. *JAMA Network Open.*

Patients were then asked if they would accept to be approached to discuss this study by their clinician. Those who agreed were approached by a certified research assistant, who provided study details and sought informed consent. Enrolled patients were then randomly assigned to receive either a curated music intervention or usual care (no music; control group) during their six sub-anesthetic ketamine infusions in a 1:1 ratio. Enrolment stopped when the predefined sample size was obtained. The randomization sequence code was stored securely by the hospital staff for the duration of the trial.

Randomization and blinding

Participants who provided consent for participation were randomized in a 1:1 simple fashion by an independent hospital staff using the computer program Research Randomizer (Version 4.0).[4] All participants received a phone call from a research assistant to inform them about their arm allocation.

Blinding participants was impossible due to the nature of the intervention. The trial's investigators, psychaitric outcome assessors and the statisticians were blinded to participants' allocation.

Assessment of the primary efficacy outcome

Hemodynamic evaluations were taken at each infusion. Blood pressure change in systolic blood pressure (SBP) at 40 minutes versus at 0 minutes between intervention and control groups was assessed by a validated and calibrated Welch Allyn Blood Pressure Device according to Hypertension Canada Guidelines and recommended technique for automated office blood pressure.[4] The patient were in a semi-reclined position (approximately 45 degrees) on the treatment bed or medical lounger, the back supported, the arm bare and supported, legs uncrossed, not talking or moving before or during the measurement, using a cuff size appropriate for the arm (i.e bladder width close to 40% of the arm circumference and length cover 80-100% of the arm circumference), the middle of the cuff at heart level, with the lower edge of the cuff 3 cm above the elbow crease. The initial measurement was taken after 10 minutes of quiet rest. No change in bed/recliner position were permitted during or between ketamine infusions. BP values (at 0 minutes and 40 minutes) were the average of the triplicate measurement taken at 1 minute interval at the 2 time-points, repeated at each infusion. BP was also recorded at 15-minute and 30-minute timepoints.

Study intervention and control groups

The ketamine treatment procedure received by both the intervention and control groups consisted of six infusions given over four weeks. During the first two weeks, infusions were given bi-weekly and weekly during the last two weeks.

Patients received their ketamine infusions in a quiet room, while positioned in a semi-reclined position on a hospital bed or medical recliner at approximately 45 degrees. The infusion was prepared by the treating team's nurse, who diluted 0.5mg/kg of ketamine in 250mL of normal saline, based on clinical information such as weight, height, and BMI. The infusion was verified

© 2024 Greenway KT et al. *JAMA Network Open.*

by another member of the treating team before being administered continuously for 40 minutes. For patients with a BMI greater than 30, the ketamine dose was calculated based on the upper-limit BMI of 30 due to greater observed hemodynamic changes in patients with a BMI of 30 and above.[5]

Following the setup of infusion equipment, patients were given 10 minutes to rest in a semi-reclined position without using any electronic devices, to allow for an accurate baseline measurement of blood pressure. Vital signs such as heart rate, respiratory rate, oxygen saturation, and blood pressure were measured by clinicians before the infusion, and at 15, 30, and 40 minutes. The infusion was administered in the presence of a nurse and physician who continuously assessed the patients' physiological and mental state during the infusion. Blindfolds were offered to all patients during the infusions.

Study intervention: Music during ketamine treatment

The intervention consisted of playing music via headphones during all ketamine treatments, beginning at the commencement of each infusion and ending approximately 55 minutes later. For the purposes of this study, several music playlists were created based on scientific literature regarding the use of music with hallucinogen-induced psychoactive drugs.[6] Specifically, the playlists are composed of various pieces of music of diverse genres mostly taken from published playlists used in psilocybin-assisted psychotherapy.[7] The music used in these playlists was free from understandable lyrics; i.e., no human vocals in a language that the patient understands. The specific songs and their order were chosen to create playlists that differ in style but are cohesive and follow a similar pattern: calming at the outset, then richer and more absorbing after approximately 10-15 minutes, then again calming for the last 20 minutes of the playlist (beginning around 35 minutes; five minutes before the 40-minute infusion endpoint).

On the day of each infusion, before the treatment begins, clinicians discussed music choices with participants to select amongst one of the available playlists based on their preferences, mood, and experiences with other playlists (for subsequent treatments). This may have entailed briefly listening to excerpts of the selected playlists. Generally, patients were asked to select unfamiliar music in accordance with recommendation from the psilocybin literature.[6] After the first treatment, patients were invited to request the incorporation of music of their choosing into the middle part of the playlists (i.e., ending no later than five minutes before the infusion termination). If requested, service clinicians modified one of the study playlists to include the patient's preferred song(s). Following the active treatment period, patients were sent a copy of the playlists used in their treatments.

Control condition: treatment as usual

The control condition consisted of receiving ketamine in the same settings, setup in the same way (except for any music listening equipment), with blindfolds, and with matched accompaniment relative to the intervention arm. The only difference between conditions was the use of music. As needed, participants in the control condition were provided with reassurance and basic breathing or mindfulness exercises by their clinicians to encourage relaxation during the infusions.

© 2024 Greenway KT et al. *JAMA Network Open.*

The amount of contact with clinicians were matched outside of the ketamine treatments. In lieu of choosing music collaboratively with their clinician at study visits before the ketamine infusion, patients received equivalent amount of contact with their clinicians. This time entailed further discussion of personal or psychiatric issues, responses to the treatment process thus far (for subsequent treatments), and relaxation exercises.

Statistical Analysis

For the primary outcome data analysis, we adopted the generalized linear model (GLM) to investigate the change in systolic blood pressure (SBP) at 40 minutes versus at 0 minutes between intervention and control groups.[8] We treated the difference between the average of the triplicate SBP measurements at 0 minutes and 40 minutes at each infusion as the outcomes in the GLM, adjusting for covariates such as intervention, age and sex. The generalized estimating equation (GEE) technique was used to estimate the regression coefficients, and the corresponding variances were estimated by the sandwich estimators.[9] Each of the hemodynamic outcome measurements used, namely change in SBP and DBP between 0 and 40 minutes, was taken as an outcome variable in separate GEE analyses. A Gaussian regression with an identity link for continuous outcomes was used to estimate mean differences between treatment groups, fitted with robust error estimation and an ar1 working correlation structure.[10] All GEE analyses were conducted using the "CRTgeeDR" package in R.[10] We used Inverse-probability weighting (IPW) to account for missing data and augmentation (AUG) for imbalance in covariates between treatment groups for doubly robust GEE estimations.[10] For IPW, missing data was assumed to be missing at random, with missingness associated with baseline personality disorder diagnosis and shorter duration of current depressive episode. To determine group treatment covariates for AUG, we first tested multivariable GEE models with treatment condition and each possible covariates in separate models to determine their unique influence on SBP and DBP change. Possible covariates included baseline differences between treatment arms (i.e., BMI, Education, antipsychotic/mood stabilizers, past hospitalizations) and factors associated with hemodynamic responses (i.e., age, sex, blood pressure medication). Then, factors without statistical significance were removed from final doubly robust GEE models. Analyses were based on an intent-to-treat principle, including all patients randomly assigned. A two-tailed p value <0.05 was considered to indicate statistical significance.

© 2024 Greenway KT et al. *JAMA Network Open.*

eTable 1. Baseline demographics and clinical characteristics.

	Music group	Non-music group	Total
**Sample size**	15	17	32
**Age (years), Mean (SD)**	43.1 (16.1)	48.4 (12.2)	45.9 (14.2)
**Female No. (%)**	11 (73)	12 (71)	23 (72)
**Race and ethnicity, No. (%)**			
White	13 (87)	14 (82)	27 (84)
Middle eastern	1 (7)	0 (0)	1 (3)
Hispanic	1 (7)	1 (6)	2 (6)
Black	0 (0)	1 (6)	1 (3)
**Employment status, No. (%)**			
Disability	9 (60)	9 (53)	18 (56)
Unemployed/Retired	5 (33)	5 (29)	10 (31)
Employed	1 (7)	3 (18)	4 (13)
**Relationship Status, No. (%)**			
Single	8 (53)	8 (47)	16 (50)
Divorced	2 (13)	2 (12)	4 (13)
Married/Cohabitating	5 (33)	7 (41)	12 (38)
**Education, No. (%)**			
≤ High school	0 (0)	1 (6)	1 (3)
High school or college	2 (13)	8 (47)	10 (31)
University	13 (87)	8 (47)	21 (66)
**Body Mass Index, Mean (SD)**[a]	23.2 (4.6)	29.3 (8.9)	26.4 (7.8)
**Medical History, No. (%)**			
Tobacco user	1 (7)	1 (6)	2 (6)
Cannabis user	3 (20)	2 (12)	5 (16)
Diabetes mellitus	1 (7)	3 (18)	4 (13)
Kidney impairment	0 (0)	1 (6)	1 (3)
Obstructive sleep apnea	1 (7)	3 (18)	4 (13)
**Baseline blood pressure, Mean (SD)**			
SBP	118.4 (13.7)	118.9 (17)	118.6 (15.2)
DBP	73.5 (6.4)	76.0 (9)	74.8 (7.8)
**Blood pressure medication, No. (%)**	1 (7)	2 (12)	3 (9)
**Depression severity Mean (SD)**			
MADRS	31.4 (4.3)	31.6 (8.2)	31.5 (6.5)

SD: Standard deviation
DBP: Diastolic Blood Pressure
SBP: Systolic Blood Pressure
MADRS: Montgomery–Åsberg Depression Rating Scale
[a] Calculated as weight in kilograms divided by height in meters squared.

© 2024 Greenway KT et al. *JAMA Network Open.*

eTable 2. Musical playlists employed in the study.

Title	Artist	Length
**Playlist 1 -- Classical**		
Echoes of Healing	Kathryn Toyama	6:02
Disclosed	Federico Albanese	4:51
Piano Concerto No. 5 in E-Flat Major, Op. 73: II: Adagio un poco mosso	Ludwig van Beethoven, Glenn Gould, Leopold Stokowski, American Symphony Orchestra	9:23
The Gift	Joep Beving	3:46
Komm, susser Tod BWV478	Johann Sebastian Bach, Leopold Stokowski, Symphonica Orchestra	5:56
Lux 4	Brian Eno	18:28
**Playlist 2 -- Ambient**		
In the Labyrinth Garden	Allo Die	43:17
Wawa by the Ocean	Mary Lattimore	10:29
**Playlist 3 -- Ambient Emotive**		
Recovery	Jon Hopkins	5:36
Templar	Harold Budd	9:32
Toil Theme Part 1	Brian McBride	2:30
Toil Theme Part 2	Brian McBride	2:44
Toil Theme Part 3	Brian McBride	2:27
Emerald and Stone	Brian Eno, Jon Hopkins, Leo Abrahams	2:12
Minack	The Echelon Effect	3:53
Ceres	Saariselka	17:45
Borderland Sorrows	Slow Meadow	3:44
Autmn Hill	Jon Hopkins	2:40
**Playlist 4 -- Piano Jazz**		
Peace Piece	Bill Evans	6:42
Turiya & Ramakrishna	Alice Coltrane	8:18
Cherry Blossom	Matthew Halsall	7:21
Yefikir Engurguro	Hailu Mergia	6:16
Pound For Pound	The Bad Plus	6:10
A Taste of Honey	Paul Desmond	4:26
Mother's Love	Emahoy Tsege Mariam Gebru	3:39
Tezeta	Mulatu Astatke	6:17
Whisky Story Time	Alabaster DePlume	2:20
**Playlist 5 -- Eno's Reflection**		
Reflection	Brian Eno	65:25
**Playlist 6 -- Azure**		
Subtext	John Foxx, Harold Budd	6:00
My Friend the Forest	Nils Frahm	6:09
Sleepers Beat Theme	Ben Lukas Boysen	4:37

© 2024 Greenway KT et al. *JAMA Network Open.*

Flight from the City	Johann Johannsson, Yuki Numata Resnick, Tarn Travers, Ben Russell, Clarice Jensen	6:31
Azure	Greg Haines	14:14
Spiegel im Spiegel	Arvo Part, Peter Minkler, Lura Johnson	11:56
Endings	Trevor Oswalt, East Forest	4:40
**Playlist 7 -- Chant**		
Prayer For Compassion	Olafur Arnalds, Nils Frahm	5:55
Faith's Hymn	Beautiful Chorus	6:20
Nocturne 17	Craig Armstrong	6:00
Introit Benedicta Sit	Monks Of The Abbey Of Notre Dame	3:22
Prayer For Compassion	David Darling	4:19
Stones Start Spinning	David Darling	4:16
Alleluia	Monks Of The Abbey Of Notre Dame	3:44
Darkwood 1	David Darling	2:24
Sanctum Et Immaculata	Monks Of The Abbey Of Notre Dame	3:42
Music Of A Desire	David Darling	2:49
Root Chakra	Beautiful Chorus	4:42
**Playlist 8 -- David Darling**		
The Beauty of All Things	David Darling	3:40
Music Of A Desire	David Darling	2:49
Remembering Our Mothers	David Darling	3:19
Mysterium	David Darling	4:02
Prayer	David Darling	6:02
Heaven Here On Earth	David Darling	2:20
Prayer For Compassion	David Darling	4:19
September Morn	David Darling	4:36
Stones Start Spinning	David Darling	4:16
When We Forgive	David Darling	4:03
Shoe Strings	David Darling	2:50
Cello Blue	David Darling	8:11
**Wavepaths**	Various	--

© 2024 Greenway KT et al. *JAMA Network Open.*

eReferences:

1. Kennedy SH, Lam RW, McIntyre RS, et al. Canadian Network for Mood and Anxiety Treatments (CANMAT) 2016 clinical guidelines for the management of adults with major depressive disorder: section 3. Pharmacological treatments. *The Canadian Journal of Psychiatry*. 2016;61(9):540-560.
2. Yatham LN, Kennedy SH, Parikh SV, et al. Canadian Network for Mood and Anxiety Treatments (CANMAT) and International Society for Bipolar Disorders (ISBD) 2018 guidelines for the management of patients with bipolar disorder. *Bipolar Disord*. 2018;20(2):97-170.
3. Galinowski A, Lehert P. Structural validity of MADRS during antidepressant treatment. *International Clinical Psychopharmacology*. 1995;10(3):157-161.
4. Rabi DM, McBrien KA, Sapir-Pichhadze R, et al. Hypertension Canada’s 2020 comprehensive guidelines for the prevention, diagnosis, risk assessment, and treatment of hypertension in adults and children. *Canadian Journal of Cardiology*. 2020;36(5):596-624.
5. Wan LB, Levitch CF, Perez AM, et al. Ketamine safety and tolerability in clinical trials for treatment-resistant depression. *J Clin Psychiatry*. Mar 2015;76(3):247-52. doi:10.4088/JCP.13m08852
6. Johnson MW, Richards WA, Griffiths RR. Human hallucinogen research: guidelines for safety. *Journal of psychopharmacology*. 2008;22(6):603-620.
7. Davis AK, Barrett FS, May DG, et al. Effects of psilocybin-assisted therapy on major depressive disorder: a randomized clinical trial. *JAMA Psychiatry*. 2021;78(5):481-489.
8. McCullagh P. *Generalized linear models*. Routledge; 2019.
9. Burton P, Gurrin L, Sly P. Extending the simple linear regression model to account for correlated responses: an introduction to generalized estimating equations and multi-level mixed modelling. *Statistics in medicine*. 1998;17(11):1261-1291.
10. Prague M, Wang R, Stephens A, Tchetgen Tchetgen E, DeGruttola V. Accounting for interactions and complex inter-subject dependency in estimating treatment effect in cluster-randomized trials with missing outcomes. *Biometrics*. 2016;72(4):1066-1077.

© 2024 Greenway KT et al. *JAMA Network Open.*
