# Supplementary material for: Music as an Intervention to Improve the Hemodynamic Response of Ketamine in Depression: A Randomized Clinical Trial
Source: JAMA Netw Open. 2024 Feb 5;7(2):e2354719. doi: 10.1001/jamanetworkopen.2023.54719 (PMC10845001; doi:10.1001/jamanetworkopen.2023.54719)
Supplement: Supplement 3. — Data Sharing Statement [file jamanetwopen-e2354719-s003.pdf]

Data Sharing Statement

Greenway. Music as an Intervention to Improve the Hemodynamic Response of Ketamine in Depression. *JAMA Netw Open*. Published February 05, 2024.
doi:10.1001/jamanetworkopen.2023.54719

Data
Data available: Yes
Data types: Deidentified participant data, Data dictionary
How to access data: Deidentified participant data and the data dictionary will be made available for reasonable requests from researchers for scientific purposes addressed to the corresponding author, kyle.greenway@mcgill.ca.
When available: With publication

Supporting Documents
Document types: None

Additional Information
Who can access the data: Deidentified participant data and the data dictionary will be made available for reasonable requests from researchers for scientific purposes, addressed to the corresponding author, kyle.greenway@mcgill.ca.
Types of analyses: For any reasonable scientific purpose.
Mechanisms of data availability: Data will be made available after the approval of a reasonable scientific proposal by the manuscript's co-authors. Depending on the nature of the request, a signed data access agreement may be necessary.
Any additional restrictions: Projects that carry significant potential to identify participants, such as database-linking projects, will not be approved.
